# A metabolism-related gene signature for predicting the prognosis in thyroid carcinoma

**DOI:** 10.3389/fgene.2022.972950

**Published:** 2023-01-04

**Authors:** Qiujing Du, Ruhao Zhou, Heng Wang, Qian Li, Qi Yan, Wenjiao Dang, Jianjin Guo

**Affiliations:** ^1^ Department of General Medicine, Third Hospital of Shanxi Medical University, Shanxi Bethune Hospital, Shanxi Academy of Medical Sciences, Tongji Shanxi Hospital, Taiyuan, China; ^2^ Department of Orthopedics, Shanxi Key Laboratory of Bone and Soft Tissue Injury Repair, Second Clinical Medical College, Shanxi Medical University, Taiyuan, China; ^3^ Department of Vascular Surgery, Second Clinical Medical College, Shanxi Medical University, Taiyuan, China; ^4^ Basic Medical College, Shanxi Medical University, Jinzhong, China; ^5^ Department of Endocrinology, Second Clinical Medical College, Shanxi Medical University, Taiyuan, China

**Keywords:** thyroid cancer, metabolism genes, survival prognosis, immune cell infiltration, immune checkpoint

## Abstract

Metabolic reprogramming is one of the cancer hallmarks, important for the survival of malignant cells. We investigated the prognostic value of genes associated with metabolism in thyroid carcinoma (THCA). A prognostic risk model of metabolism-related genes (MRGs) was built and tested based on datasets in The Cancer Genome Atlas (TCGA), with univariate Cox regression analysis, LASSO, and multivariate Cox regression analysis. We used Kaplan-Meier (KM) curves, time-dependent receiver operating characteristic curves (ROC), a nomogram, concordance index (C-index) and restricted mean survival (RMS) to assess the performance of the risk model, indicating the splendid predictive performance. We established a three-gene risk model related to metabolism, consisting of *PAPSS2*, *ITPKA*, and *CYP1A1*. The correlation analysis in patients with different risk statuses involved immune infiltration, mutation and therapeutic reaction. We also performed pan-cancer analyses of model genes to predict the mutational value in various cancers. Our metabolism-related risk model had a powerful predictive capability in the prognosis of THCA. This research will provide the fundamental data for further development of prognostic markers and individualized therapy in THCA.

## Introduction

Thyroid carcinoma (THCA), the most common endocrine malignancy, affects approximately 65,000 patients in the United States yearly, with an increasing incidence (Mady et al., 2020). It accounts for 3.1 percent of cancer incidence worldwide (Gallardo et al., 2020). It is evaluated that the number of new cases of thyroid carcinoma is over 213000, and 35000 patients succumb to thyroid carcinoma yearly (Wang et al., 2019). There are four types of thyroid carcinoma based on histological characteristics, which consist of medullary follicular thyroid cancer (FTC), thyroid cancer (MTC), papillary thyroid cancer (PTC), and anaplastic thyroid cancer (ATC). Healing with standard therapy is difficult for some DTCs (<10%), many MTCs and almost all ATCs, but with a result of spreading to distant metastatic sites (Naoum et al., 2018). Therefore, targeted and immune therapies are receiving increasing attention showing a significant clinical value in preclinical and clinical studies (Laha et al., 2020). For this reason, there is imperative to create innovative prognostic models or acquire atypical biological markers to make targeted therapies more achievable and advance the survival of patients with TC.

Metabolic reprogramming, a symbol of cancer, empowers malignant cells’ potential for growth and diffusion in the tumor microenvironment (TME) that is short of nutrition, and the favorable changes for cancer cells are initiated by the synthetic effects of oncogenic alterations in host cell factors and malignant cells (Dey et al., 2021). Research carried out by advanced survival analysis displays that enhanced overall metabolism-related gene expression in tumors always leads to unsatisfactory survival outcomes in 33 cancer types (Zhang et al., 2020). Recent studies have investigated the prognostic models of MRGs in several cancers, like lung adenocarcinoma (Zhang et al., 2021a), Ewing’s sarcoma (Fu et al., 2021), and stomach adenocarcinoma (Ye et al., 2021). We hope to build a risk model about metabolism-related genes to help predict clinical prognosis and precise treatment by combining it with the current status of thyroid carcinoma treatment and the frontiers of metabolism in the field of cancer. In this research, we integrated the expression profile of MRGS in patients with thyroid carcinoma and obtained the metabolic genes associated with prognosis in combination with survival data. A prognostic risk model of MRGs, validated in the test cohort, was constructed through various advanced statistical methods. With calculated risk scores of patients, the two sets were grouped into high-stakes and low-stakes cohorts. Therefore, we can observe differential genes associated with metabolism between tumor and normal examples, functional enrichment, immune infiltration, mutation, and therapeutic reaction in different risk groups, thereby providing references for individualized treatment of patients at different risk states.

## Materials and methods

### The acquisition of data

We transferred 570 gene expression profiles of THCA From Harmonized Cancer Datasets, which was made of 58 normal and 512 tumor samples. The Clinical features of 615 samples from the TCGA database were transferred from the UCSC Xena website (https://xena.ucsc.edu/). From the Molecular Signatures Database, we got the key file (c2.cp.kegg.v7.4.symbols.gmt) to find 944 MRGs, and we extracted MRGs expression from the expression data. All THCA gene expression data had been transformed into log2 (x+1) and we took the average of the same gene expression value.

### The screening of differentially expressed genes

We selected Differentially Expressed Genes (DEGs) tied with metabolism between tumor and normal patients using the “limma” package in R language (version 4.1.0). With the statistical method of the Wilcox test, FDR of <0.05 and | logFC | of >0.5 were formulated as the screening criterion. The final differential results were displayed in heatmap and volcano plot.

### Functional enrichment of DEGs

In what followed, we annotated DEGs using Gene Ontology (GO) enrichment analysis and Kyoto Encyclopedia of Genes and Genomes (KEGG) pathway analysis *via* the “clusterProfiler” package in R language and then visualized the results of enrichment analysis using “enrichplot” and “ggplot2″ packages.

### Construction of metabolism-related prognostic model and survival analysis

All thyroid cancer patients were randomly divided into a training set (n = 252) and a test set (n = 248) at a one-to-one ratio. The training set was used to identify prognostic metabolism-related genes and develop a prognostic risk model, which was then validated in the test set. Clinical characteristics were short of significant differences in the two cohorts (Table 1). Univariate Cox regression analysis (*p* < 0.01) was employed to identify the metabolic-related DEGs related to patient survival initially. Subsequently, LASSO Cox regression analysis (Tibshirani, 1997), first proposed by Robert Tibshirani in 1996 and a commonly used regularization method for automatic feature screening, was used to eliminate the highly correlated genes to avoid the over-fitting of the model with the R package “glmnet”. Finally, we conducted a multivariate Cox regression analysis to construct the ideal prognostic mode, using the genes selected by LASSO. Immunohistochemistry (IHC) verification data was caught from the Human Protein Atlas (HPA) database (https://www.proteinatlas.org/). After that, we reckoned the patients’ risk scores in two sets according to the risk score formula of the established prognostic model. To endorse the model’s forecasting capability, we parted the patients of two sets into a high-risk cohort and low-risk cohort separately to plot survival curves and ROC curves *via* the “survival” package, “survminer” package and “timeROC” package, with the median risk score of the training group as the segmentation value. Principal Component Analysis (PCA) in three dimensions and t-distributed Stochastic Neighbor Embedding (t-SNE) using a Barnes-Hut Implementation were carried out with the “scatterplot3d” and “Rtsne” packages, respectively. A nomogram was created using the nomogram function from the “rms” package. As an indicator to appraise the prediction accuracy, we calculated the concordance index (c-index) and restricted mean survival (RMS) of other models (Pak et al., 2019; Wu et al., 2019; Han et al., 2020; Lv et al., 2020; Ren et al., 2021; Wen et al., 2021; Zhen et al., 2021) and the model we developed. Gene Set Enrichment Analysis (GSEA) was carried out between high-stakes and low-stakes cohorts *via* GSEA 4.2.2 (Subramanian et al., 2005).

**TABLE 1:** The clinical features in training and test sets.

Variables	Type	Training set (n = 252)	Test set (n = 248)	Sig
Age	≤60	192	196	0.446
	>60	60	52	
Gender	Female	192	173	0.106
	male	60	75	
T	T1	68	73	0.979
	T2	89	75	
	T3	82	88	
	T4	12	11	
	unknown	1	1	
N	N0	114	114	0.887
	N1	113	110	
	unknown	25	24	
M	M0	151	130	0.063
	M1	2	7	
	unknown	99	111	
Stage	Stage I	140	141	0.848
	Stage II	58	25	
	Stage III	27	54	
	Stage IV	27	28	

### Construction of correlation network

Genes were used to conduct the correlation network *via* the “igraph” and “reshape2″ packages, with 0.2 as the correlation coefficient threshold, after being selected by Univariate Cox regression analysis (*p* < 0.05). We also analyzed the transcription factors associated with these genes by Pearson Correlation analysis (*p* < 0.001; coefficient >0.5), with the result displayed with a Sankey Diagram using the “ggalluvial” package.

### Independent prognostic value of the risk score achieved from the metabolism-related prognostic model

We conducted a univariate Cox regression analysis, Log-rank test, and Breslow test (*p* < 0.05) to evaluate the prognostic values of clinical characteristics and risk scores in the training set. Furthermore, multivariate prognostic analysis for the training set (*p* < 0.05) was carried out to assess whether the risk score achieved from the metabolism-related model could be an independent predictor. In addition, we investigated the link between clinical characteristics and risk scores through the statistics software SPSS 26.0. In addition, considering the pathological subtypes of thyroid cancer, we conducted a differential analysis of risk scores for different pathological subtypes *via* analysis of variance (ANOVA). Simultaneously, we performed multiple linear regressions to discuss the effect of different pathological types on the risk score.

### Immunogenomic landscape analyses of the metabolism-related prognostic model

We analyzed the immunogenomic landscape to determine the link between the THCA metabolism-related prognosis Model and immune status. The overall immune infiltration in the training and test set and the survival difference of different immune cells in the high-stakes and the low-stakes groups were evaluated with CIBERSORT algorithms. The immune-related functions were compared between the high-stakes and the low-stakes cohorts with single-sample gene set enrichment analysis (ssGSEA) algorithms using the “GSVA” package, “limma” package, and “GSEABase” package. We assessed the correlation between risk score and TME-related score with ESTIMATE algorithms, visualized using the “ggpubr” package. Furthermore, we investigated the expression difference of common immune checkpoints in the high-risk and low-risk cohorts.

### Drug susceptibility analysis

We used the “pRRophetic” R package to forecast the half-maximal inhibitory concentration (IC_50_) of antitumor drugs that had statistical significance between two risk cohorts in the training set for exploring the clinical value of risk models for antitumor therapy in thyroid cancer patients. Subsequently, we took advantage of the CellMiner database (https://discover.nci.nih.gov/cellminer/home.do) to get drug sensitivity data and gene expression data, then chose drugs that FDA approved, performing Pearson correlation analysis (“co. Test” function) between model genes expression and drug response. In order to explore the radioactive iodine treatment response in different risk cohorts, we performed differential analysis and correlation analysis of NIS gene expression.

### Mutation analysis

Mutation data from TCGA were transformed into the MAF file. The application of “maftools” package presented the mutation landscape in the waterfall plots. The variance of tumor mutation bearing (TMB) calculated from the MAF file was analyzed using the Wilcoxon test.

### Pan-cancer analyses related to mutation of model genes

To inquire into the expression and mutation of model genes in different cancers, the expression of model genes in pan-cancer was examined using Tumor Immune Estimation Resource (TIMER) web address (Li et al., 2017). Moreover, we explored TMB and Microsatellite Instability (MSI) of model genes in pan-cancer, displayed as radar charts *via* the “fmsb” package.

### Cell culture

The B-CPAP cell line (human thyroid papillary carcinoma cells) was purchased from the Chinese Academy of Sciences Cell Bank (Shanghai, China). As a normal control, the Nthy-ori 3–1 cell line (SV-40 immortalized normal human thyroid epithelial cells) was purchased from Sunncell Biological Company (Wuhan, China). The *mycoplasma* test result of the cell line was negative, and the STR test was correct. All cell lines were nurtured in RPMI-1640 medium mixed with 10% fetal bovine serum, in 5% carbon dioxide, at 37°C.

### The qRT-PCR validation of model genes

Total RNAs of cells were collected using the M5 universal RNA Mini Kit Tissue/Cell RNA Rapid Extraction Kit (Mei5 Biological Company, Beijing, China). The PrimeScript RT Master Mix (Perfect Real Time) (Takara Bio Inc., Beijing, China) and C1000 Touch Thermal Cycler (Bio-Rad, Hercules, CA, United States) were used for reverse transcription. qRT-PCR was carried out with 2X M5 HiPer Realtime PCR Super mix with Low Rox (Mei5 Biological Company, Beijing, China) and QuantStudio 6 Flex Real-Time PCR System (Thermo Fisher Scientific Incorporated, Waltham, MA, United States). ACTB gene was used as the internal control for normalization of target mRNA levels. The relative expression of model genes (*PAPSS2*, *ITPKA* and *CYP1A1*) was computed using the 2^−ΔΔCt^ method. We gave a summary of the sequences of the primers in Supplementary Table S1.

### Immunofluorescent staining

On 12-well plates, cells were seeded and fixed for 30 min with ice-cold paraformaldehyde. To block non-specific staining, we incubated cells with sheep serum after washing them with PBS. Cells were incubated overnight with primary antibodies at 4°C and secondary antibody (GB21303, 1:300, Servicebio, China) for 1 h. Here are the primary antibodies we used: CYP1A1 (PB9544, 1:200, Boster, China), ITPKA (14270-1-AP, 1:100, Proteintech, China), PAPSS2 (CSB-PA527262LA01HU, 1:100, Cusabio, China). Finally, DAPI (AR1176, Boster, China) was used to observe the nucleus. Fluorescence microscopy was used to observe the staining.

### Statistical analysis

We used Strawberry Perl software (version 5.30.1-64bit) and R software (version 4.1.0) to analyze all statistics. In some cases, we used SPSS software (SPSS 26.0, SPSS Inc.) and GraphPad Prism Software (version 8.3 for Windows, GraphPad). The interpretation of the *p*-value was in line with medical statistics.

## Results

### Identification and analyses of differentially expressed MRGs

**FIGURE 1:**
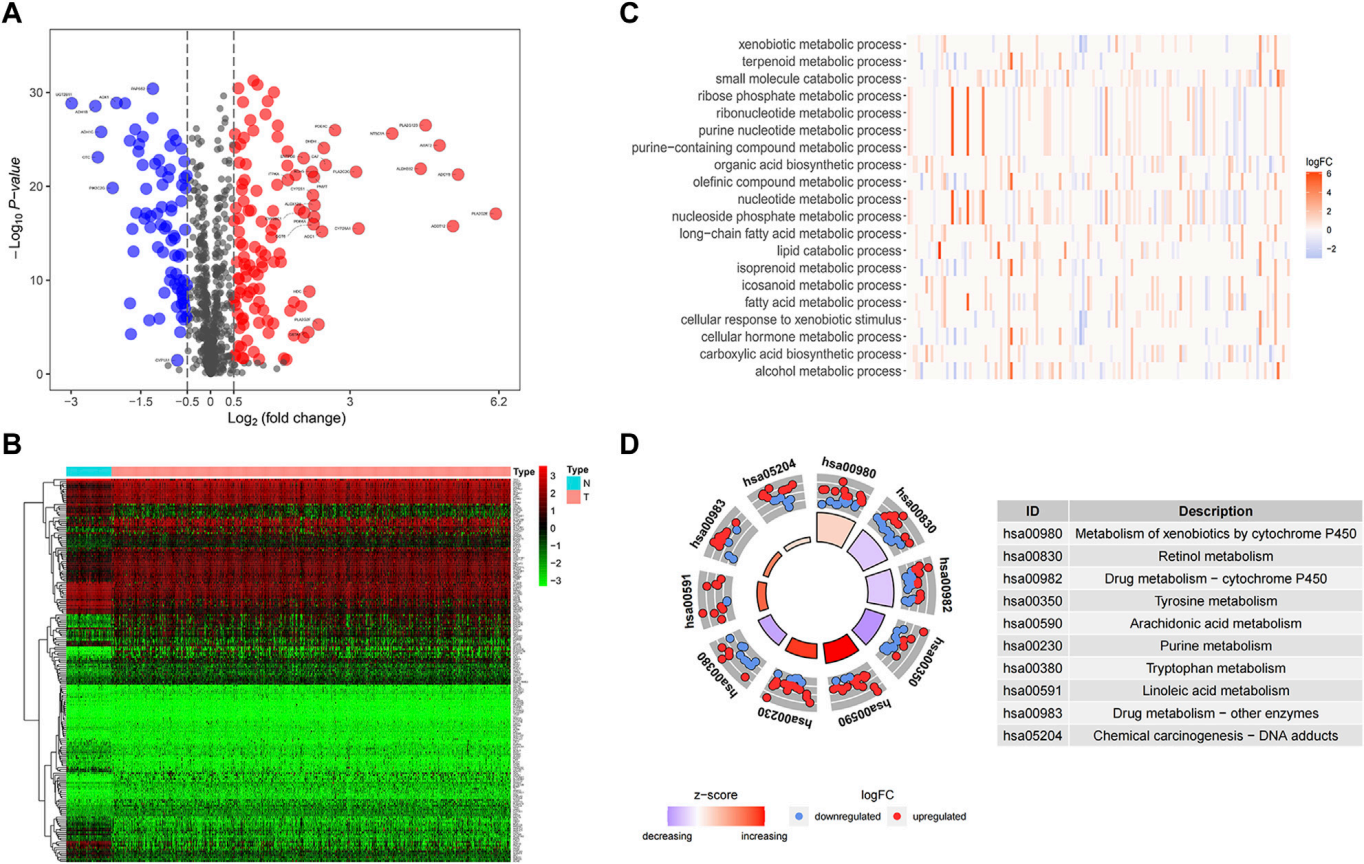
Identification and functional enrichment of differentially expressed genes. **(A)** A volcano map of differential expression genes showed 120 upregulated and 81 downregulated genes. Red represents upward regulation in tumor tissue, while blue represents downward regulation. PAPSS2, CYP1A1, and ITPKA were labeled in the figure to indicate their expression trends in tumor samples. In addition, genes with prognostic significance and | logFC | greater than 2 were specifically marked. **(B)** A heat map diagram of differential gene expression in tumors and normal samples. **(C)** The significant GO terms were depicted as a heat map with color annotation of up-regulated or down-regulated genes. **(D)** The results of KEGG enrichment analysis were demonstrated in the circle diagram, each trapezoid representing a KEGG pathway, where red and blue dots represented up-regulated and downregulated genes respectively.

After analyzing differential expression of 944 MRGs on normal (n = 58) and tumor (n = 512) groups in TCGA-THCA dataset (|logFC| > 0.5, FDR <0.05), we found 120 upregulated and 81 downregulated genes (Figures 1A,B). Functional enrichment analysis provided a biological understanding of these metabolism-associated DEGs in THCA. GO enrichment analysis demonstrated biological processes with the most significant enrichment, comprising nucleoside phosphate metabolic process, small molecule catabolic process, and olefinic compound metabolic process (Figure 1C). KEGG enrichment analysis depicted that differentially expressed MRGs mainly contained pathways in the metabolism of xenobiotics by cytochrome P450, retinol metabolism, tyrosine metabolism, purine metabolism, linoleic acid metabolism and so on (Figure 1D).

### Prognostic model construction based on MRGs

**FIGURE 2:**
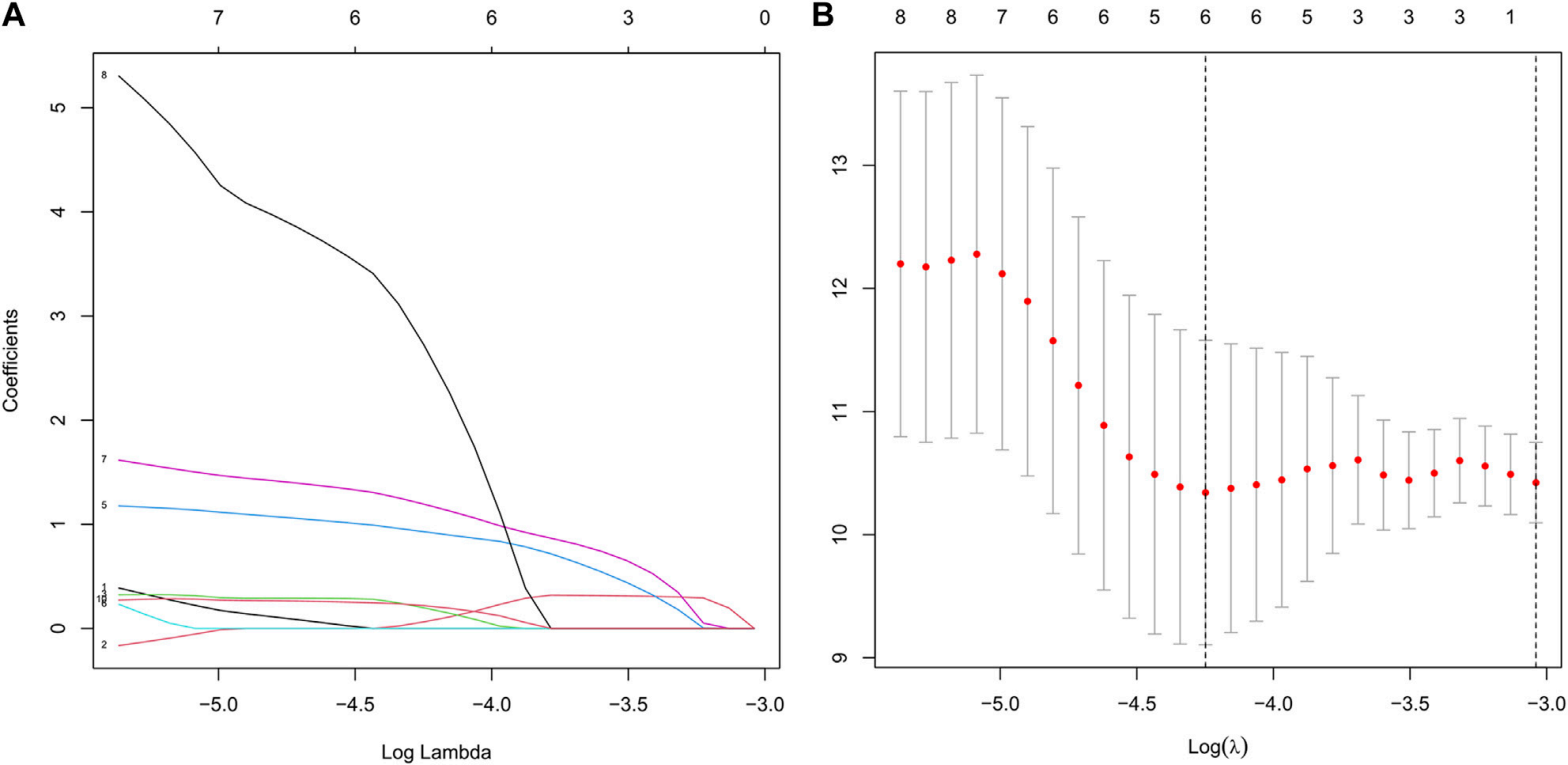
Establishment of the risk model based on LASSO Cox regression analysis and multivariate Cox regression analysis. **(A)** We chose the optimal penalty parameter lambda in line with the point of a minimum validation error. **(B)** In line with the optimal penalty parameter lambda, the metabolism-related genes with non-zero coefficient were incorporated for the following model establishment.

**FIGURE 3:**
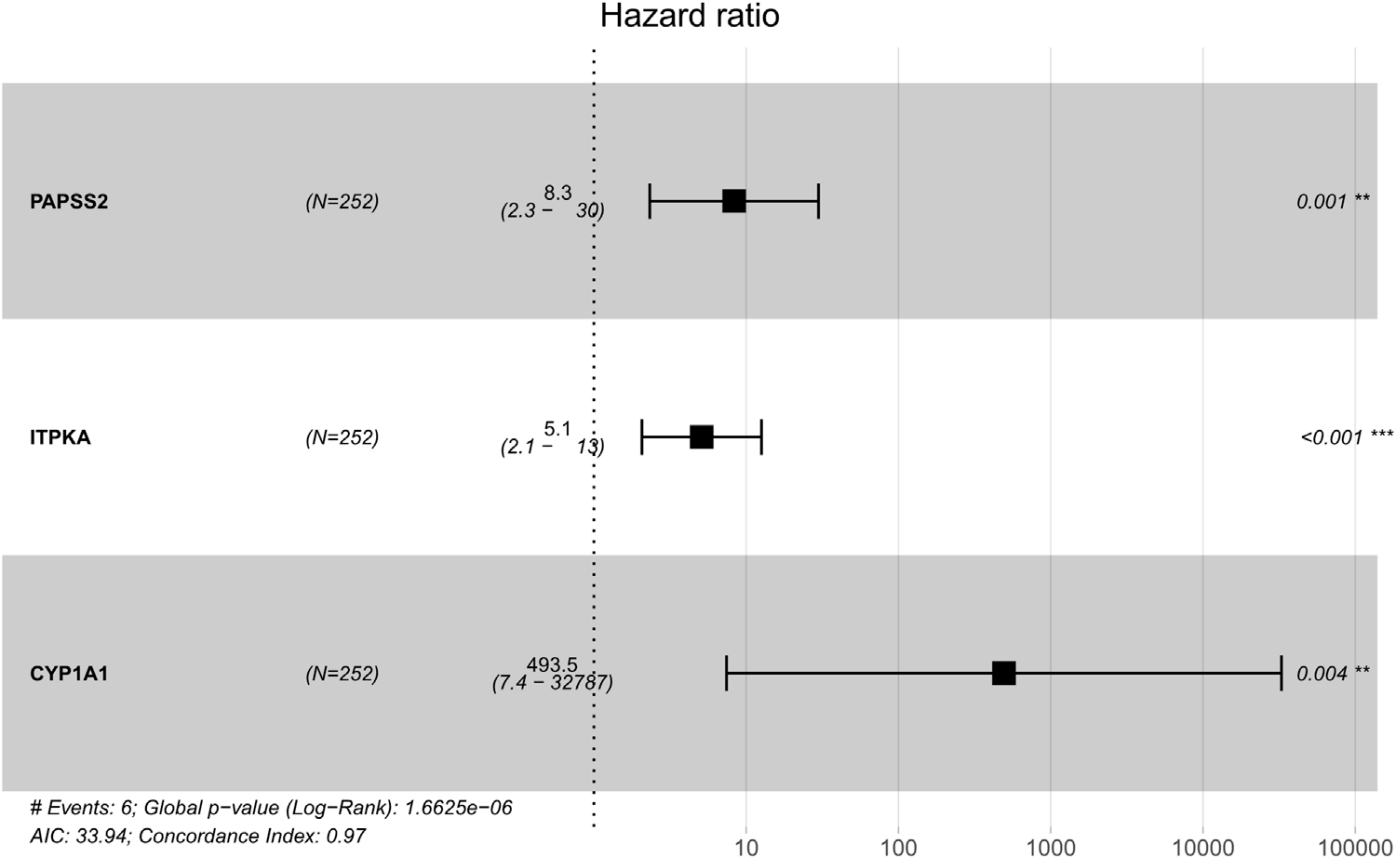
Multivariate Cox regression analysis demonstrated hazard ratio and 95% confidence intervals for three model genes.

**FIGURE 4:**
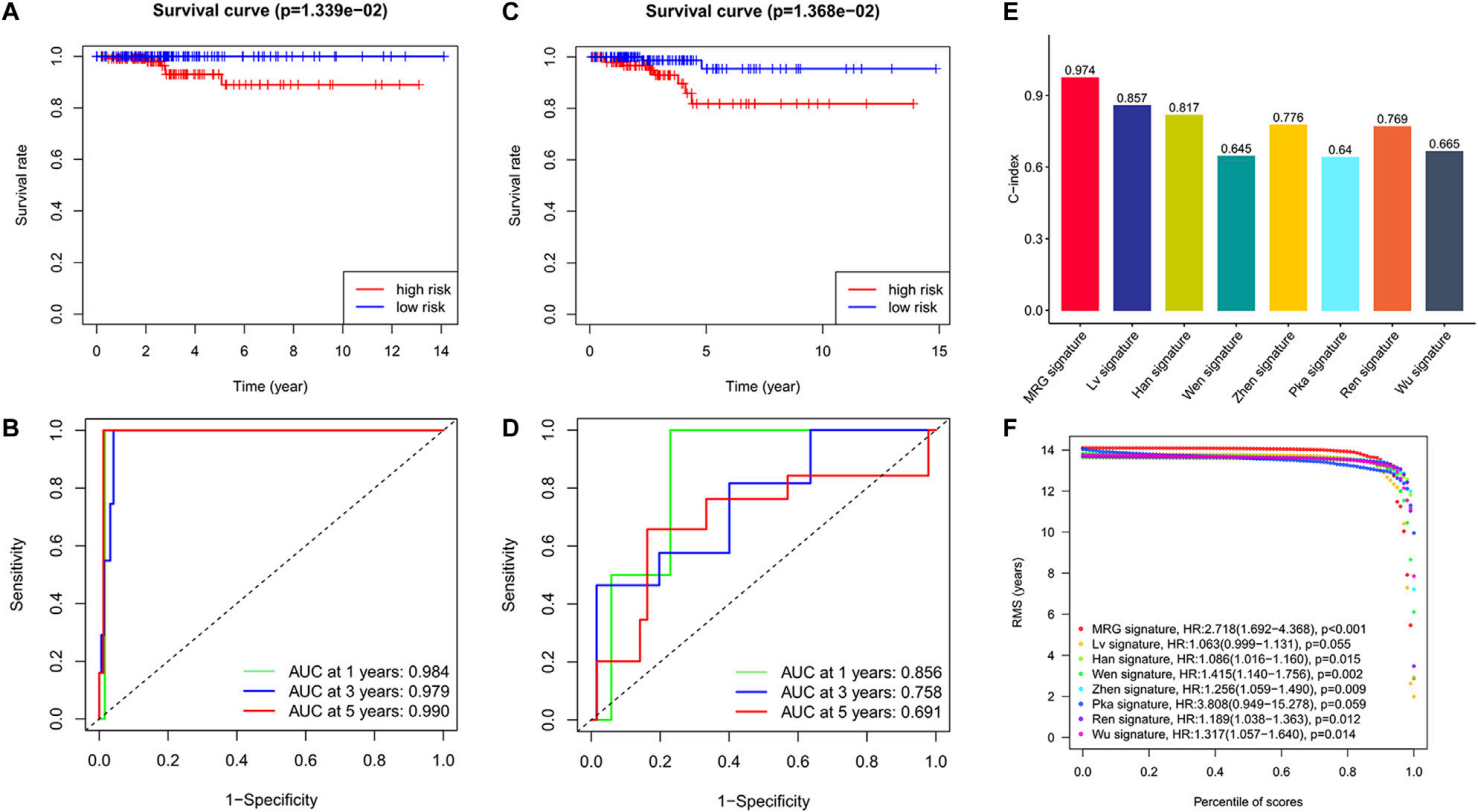
Excellent predictive performance of MRG model we developed. **(A)** KM survival curves for OS between high and low-stakes groups in the training cohort. **(B)** KM survival curves for OS between high and low-stakes groups in the test cohort. **(C)** ROC curve in the training cohort. **(D)** ROC curve in the test cohort. **(E)** The comparison of C-index among risk models. **(F)** The observation of RMS among risk models.

**FIGURE 5:**
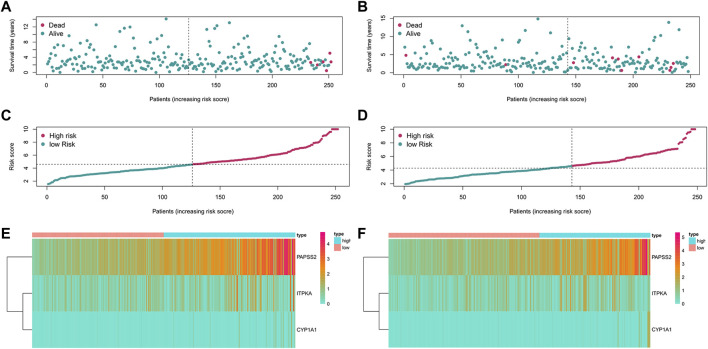
Risk curves, dot plots and heatmaps in training and test set. **(A)** The distributions of patients in training set in the risk curve. **(B)** The distributions of patients in the test set in the risk curve. **(C)** The survival status of patients in training set in the dot plot. **(D)** The survival status of patients in the test set in the dot plot. **(E)** Model gene expression of patients in training set. **(F)** Model gene expression of patients in test set.

Based on patients’ survival data in the TCGA-THCA training set, 201 DEGs related to THCA metabolism were included in a univariate Cox regression analysis. *p* < 0.01 determined that ten genes that met the screening criteria were identified as the threshold for filtering. Increasing selection methods played their respective advantages in creating gene signatures (Pak et al., 2020), but LASSO was easier to interpret than other methods (Zhang et al., 2017), and it has also been applied for the construction of many prediction models recently. With ten-round cross-validation for the optimal penalty parameter lambda, six prognostic MRGs were picked up (Figure 2). After the stepwise multivariate Cox regression analysis, three metabolic genes eventually accomplished the prognostic model establishment (Figure 3). The final model formula was that each patient’s risk score = 2.121*PAPSS2 + 1.630*ITPKA + 6.202*CYP1A1, which meant the sum of the products of the expression level of each model gene and their respective coefficients. A nomogram was created to demonstrate the predictive performance of the model better (Supplementary Figure S1). Then we corroborated the expression of model genes based on IHC data in the HPA database (Supplementary Figure S2). For succeeding survival analysis, we divided patients into two sets of high-stakes and low-stakes groups, all samples’ risk scores reckoned. The consequences of PCA in three dimensions and t-SNE affirmed the precision of the assortment among the thyroid carcinoma samples (Supplementary Figure S3). The survival curves between high-stakes and low-stakes cohorts were different (*p* = 0.013), and the survival rate of the low-stakes cohort was better apparently in contrast with the high-stakes cohort (Figure 4A), which could also be checked in the test set (Figure 4B). We made receiver operating characteristic (ROC) curve analyses to further endorse the accuracy and sensitivity of the model (Figures 4C,D). Our work validated that the model could forecast the chance of survival for one-year [area under the curve (AUC) = 0.983], three-year (AUC = 0.979), and five-year (AUC = 0.983) survival time precisely and sensitively. Compared to the C-index and RMS of four published risk models, the risk model we developed has great advantages (Figures 4E,F). Sample distributions and survival conditions were displayed as risk curves and dot plots (Figures 5A–D), and model gene expression were depicted in Figures 5E,F. As a whole, this metabolism-related risk model would become a robust predictive model of thyroid carcinoma. We performed GSEA enrichment analysis between two risk groups and attained 100 KEGG pathways, with 10 pathways figured out (Supplementary Figure S4). Five pathways, which contained B cell receptor signaling pathway, pathways in cancer, primary bile acid biosynthesis, TGFβ signaling pathways, and type Ⅱ diabetes mellitus, were most remarkably enriched in high-stakes cohorts (*p* < 0.05).

### The establishment of prognosis-related genes correlation network

**FIGURE 6:**
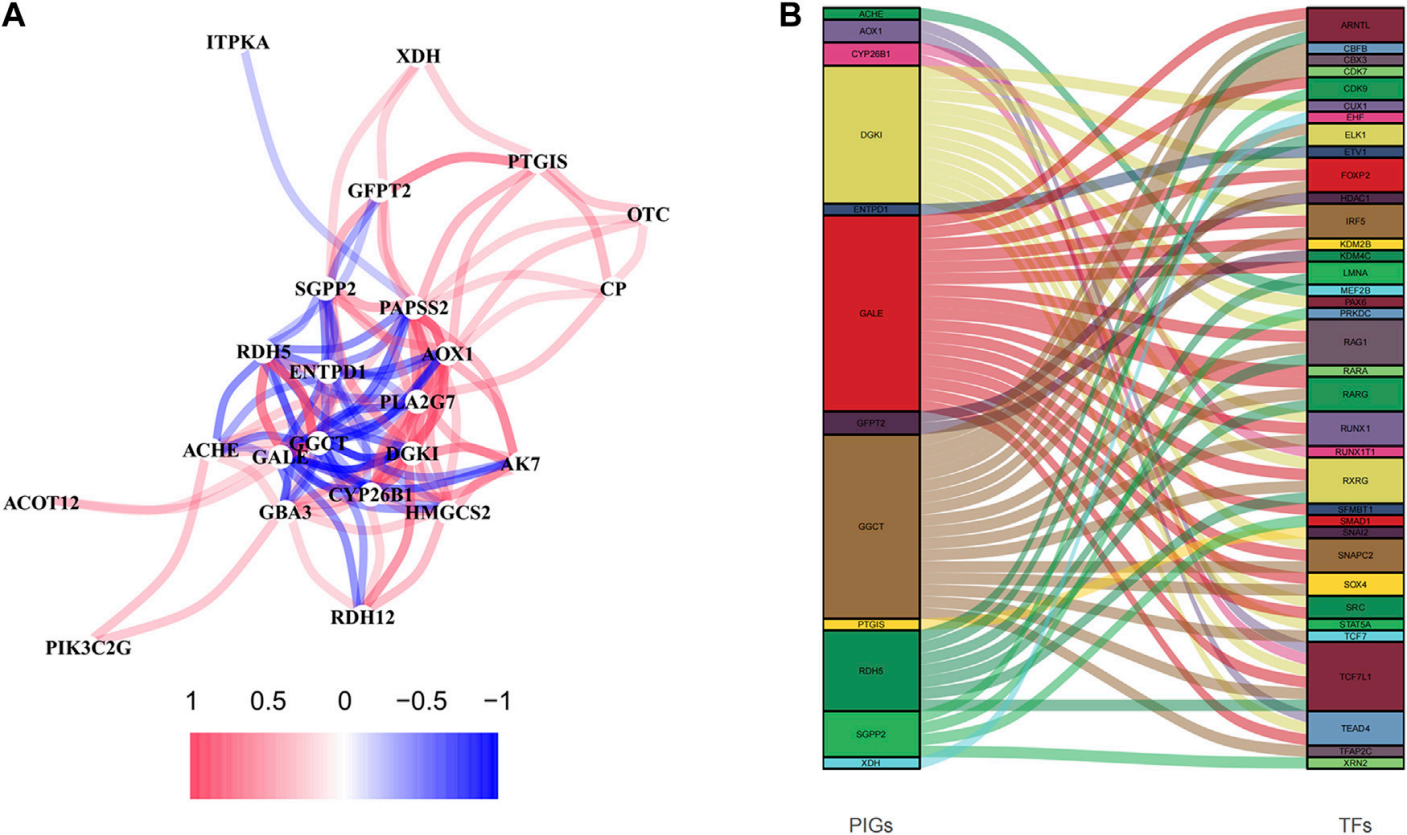
The analyses of prognosis-related genes. **(A)** The correlation network diagram was made of prognosis-related genes, 0.2 as the correlation coefficient threshold. **(B)** Correlation analysis of prognosis-related genes and transcription factors.

The network diagram displayed 25 metabolism-related genes selected using Univariate Cox regression analysis (Figure 6A), with the red lines representing positive correlation. Three genes were excluded because they did not meet the requirements of the correlation coefficient. Besides, we explored the correlations between these genes and transcription factors, demonstrated in a Sankey diagram (Figure 6B), with *p* < 0.001 and correlation coefficient >0.5 as the filtering criteria. Prognostic-related genes defined the color of the line.

### Independent prognostic value of the risk score based on three-gene metabolism-related model

**FIGURE 7:**
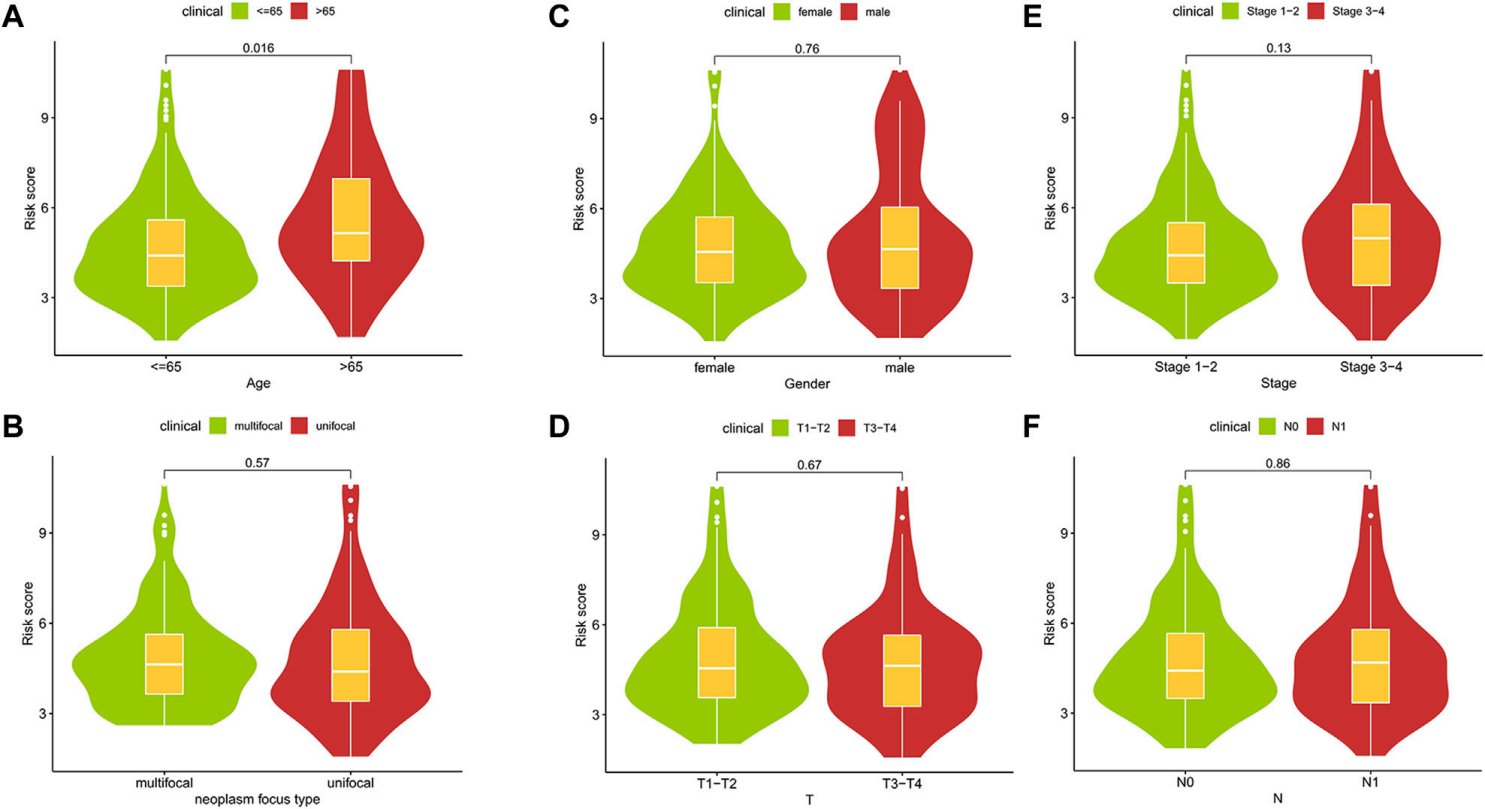
The relationships between risk scores and clinic features.

Through the different statistical methods mentioned above, we studied the value of research factors in prognostic assessment, consisting of clinical features and risk scores (Table 2). The results depicted that our risk score was competitive compared to different clinical features and can be used as an independent prognostic factor. In addition, we explored the relationship between clinical characteristics and risk scores, which demonstrated significant differences in risk scores across age groups (Figure 7). Then we described the pathological typing in the training group, and did not include anaplastic thyroid carcinoma with extremely poor prognoses (Supplementary Table S2). Risk scores among different pathological types were not statistically significant (Supplementary Table S3). Concurrently, the results of multiple linear regression indicated that different pathological types had little effect on the risk score (Supplementary Table S4).

**TABLE 2:** Application of statistical methods related to prognostic analysis in the training cohort.

Variables	Univariate ox regression analysis			Log-rank test	Breslow test
	Hazard ratio	95%CI	Sig	Sig	Sig
Age	1.159	1.060–1.267	0.001	0.000	0.001
Gender	0.279	0.056–1.394	0.120	0.097	0.171
Stage	0.211	0.039–1.156	0.073	0.048	0.055
Risk score	2.718	1.692–4.368	0.000	0.013	0.024

Variables	Multivariate analysis				
		Hazard ratio		95% CI		Sig
Age	1.209		0.979–1.493		0.077
Gender	0.708		0.110–4.578		0.717
Stage	22.252		0.198–2500.070		0.198
Risk score	2.559		1.260–5.196		0.009

### Immunogenomic landscape analyses

**FIGURE 8:**
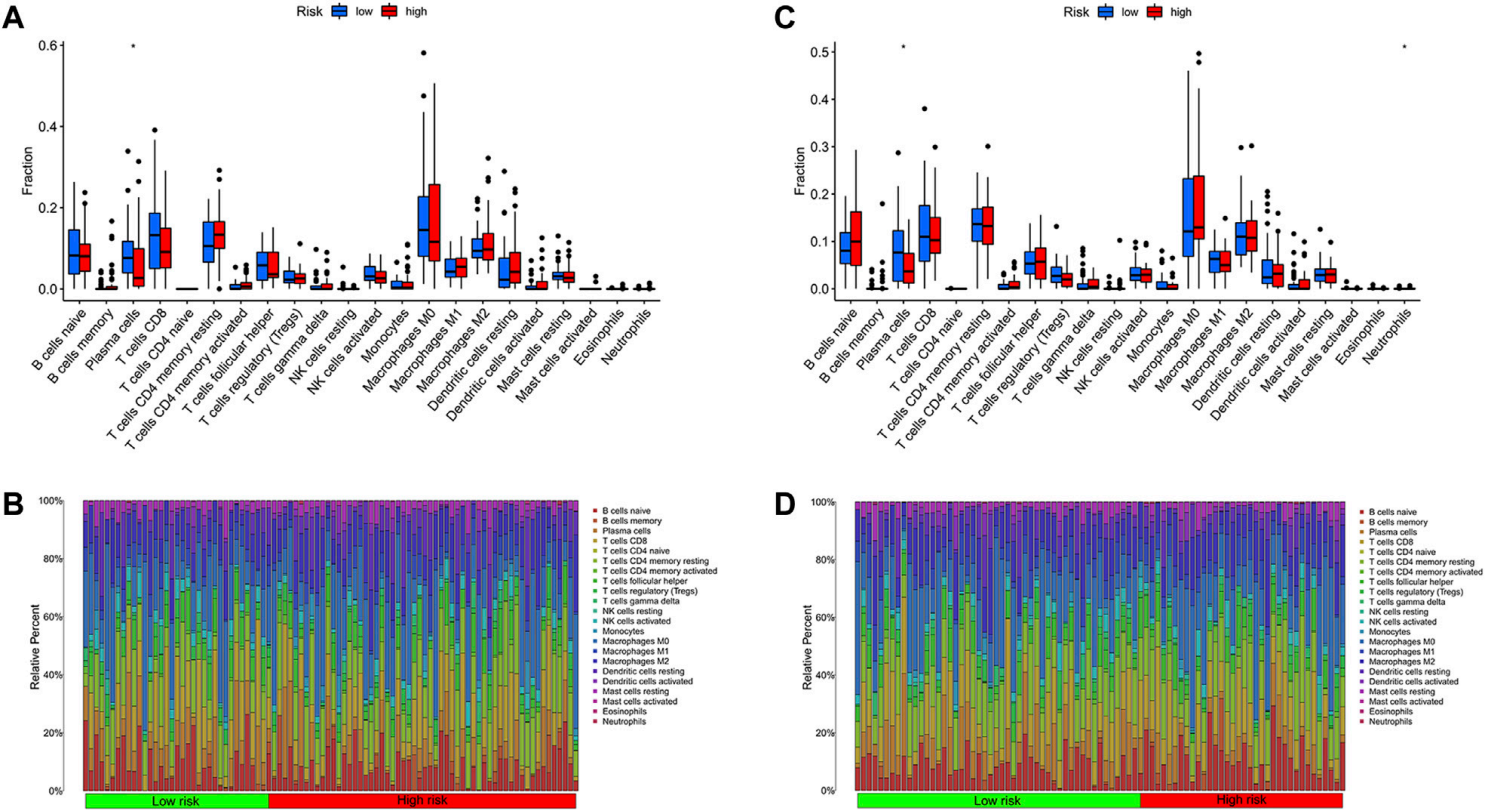
Immune cell analyses between risk cohorts predicated on CIBERSORT algorithm. **(A)** The comparison of immune cell infiltration levels in the training cohort. **(B)** Immune cell composition of patients in the training cohort. **(C)** The comparison of immune cell infiltration levels in the test cohort. **(D)** Immune cell composition of patients in the test cohort.

Some necessary indicators were assessed in different risk cohorts to investigate the link between the metabolism-related model and immune response. Using the CIBERSORT algorithm, the scores of immune cells were assessed to compare differences between groups (Figures 8B,D). The plasma cell was statistically different between risk cohorts in training and test sets (*p* < 0.05; Figures 8A,C). Furthermore, we explored the survival difference of plasma cells, which were grouped predicated on the scores of immune cells. As expected, the score of plasma cells was closely linked to the survival of patients with thyroid carcinoma (Supplementary Figure S5). In the analysis of immune-related functions, three functions of parainflammation, CCR and type II IFN response were considered statistically different from the technical assistance of ssGSEA algorithms (Supplementary Figure S6A). Through the training set and test set, the stromal score both depicted an evident rise as the risk score increased (training set: R = 0.25, *p* = 5.3E-05; test set: R = 0.24, *p* = 1.4E-04). However, based on the ESTIMATE algorithms, the immune score was not related to the risk score not as expected (Supplementary Figure S7). We analyzed expression levels of immune checkpoints of THCA in different risk groups, further assessing the availableness of the manufactured risk model in immunotherapy (Supplementary Figure S6B). According to training cohorts, *IDO1*, *IDO2*, *CD80* and *CD28* were most different statistically in the two risk groups (*p* < 0.001).

### Clinical values in antitumor therapy

**FIGURE 9:**
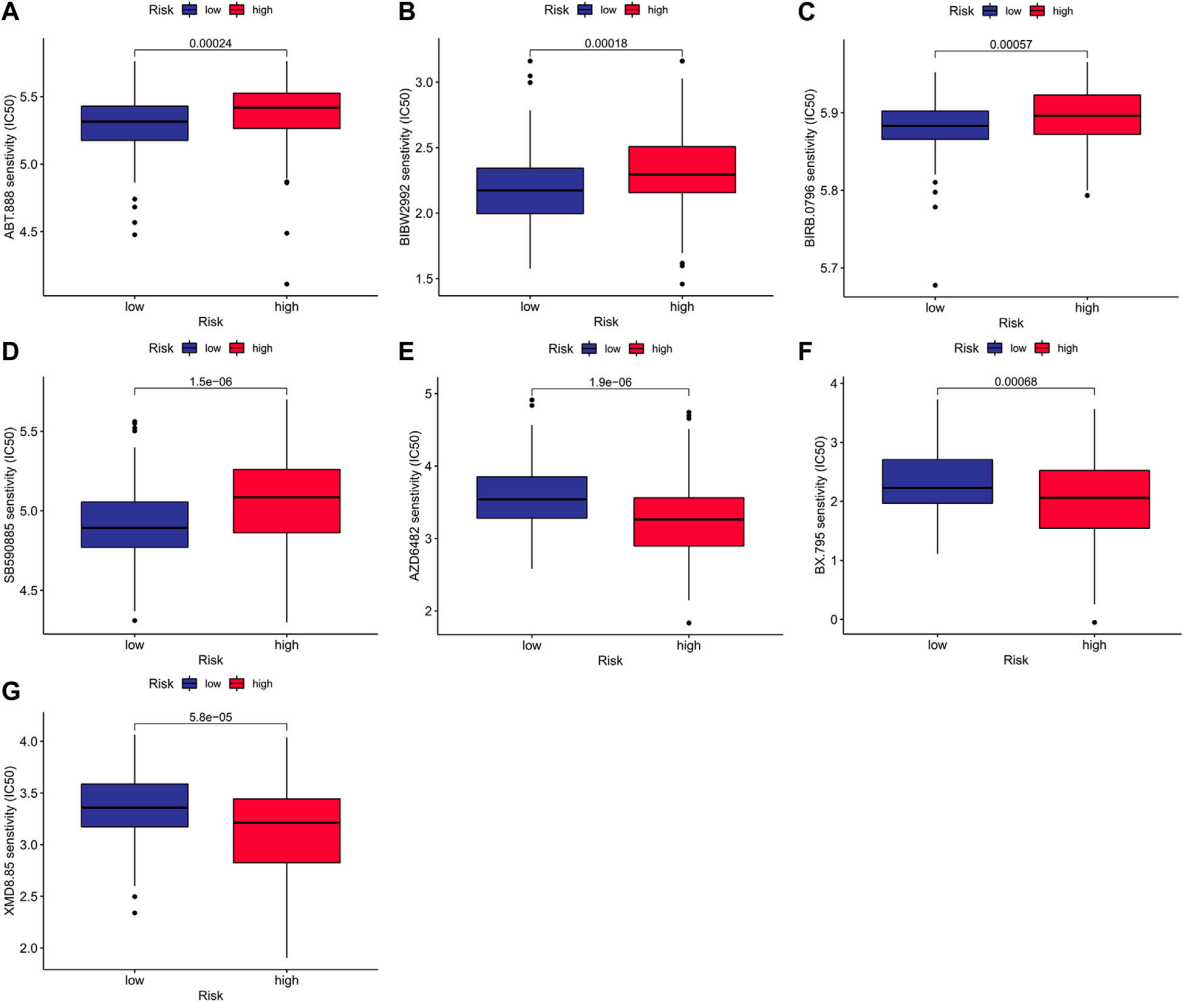
Prediction of treatment response to antitumor drugs between risk groups.

To compare the chemotherapeutic differences of risk groups, it was clear to foresee the response to antitumor drugs for treatment on the employment of IC50. A group with a smaller IC50 represents a better drug response. We found out that, Veliparib (*p* < 0.001; Figure 9A), Afatinib (*p* < 0.001; Figure 9B), Doramapimod (*p* < 0.001; Figure 9C) and SB590885(*p* < 0.001; Figure 9D) had meaningfully higher IC50 values in the high-risk cohort. In the low-risk cohort there were higher IC50 values of AZD6482 (*p* < 0.001; Figure 9E), BX-795 (*p* < 0.001; Figure 9F) and XMD8-85 (*p* < 0.001; Figure 9G). We investigated the relationship between the effect of antitumor drugs and the expression of model genes using the CellMiner databank (Supplementary Figure S8). Increased *PAPSS2* expression was linked to increased drug sensitivity of cancer cells to BLU-667, XAV-939, Staurosporine, Telatinib, and entosplenitib, whereas decreased *PAPSS2* expression was linked to decreased drug sensitivity of cancer cells to EMD-534085, Tamoxifen, ARQ-621, Pipamperone, and GNE-617. The expression of *ITPKA* went up constantly while drug sensitivity of cancer cells to Alisertib, NTRC-0066-0, SNS-314 and CCT-271850 rose. The *NIS* (*SLC5A5*) expression in the high-risk group was higher than that in the low-risk group in Supplementary Figure S9A, and there is a positive correlation between the NIS expression and risk score in Supplementary Figure S9B.

### Mutation status of the established model and model genes

**FIGURE 10:**
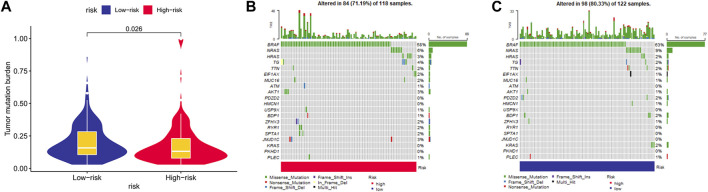
Mutation status between risk groups. **(A)** The comparison of tumor mutational burden. Mutation profile in **(B)** high-risk group and **(C)** low-risk group.

When comparing the two risk cohorts, the TMB value was significantly higher in the low-risk cohort (*p* = 0.026, Figure 10A). Waterfall plots further demonstrated frequently mutated genes in risk groups of the training cohort, revealing higher mutation frequency in the low-risk group (Figures 10B,C). BRAF and NRAS mutation frequencies were second to none in risk groups, mostly missense mutations.

### Expression levels of model genes

**FIGURE 11:**
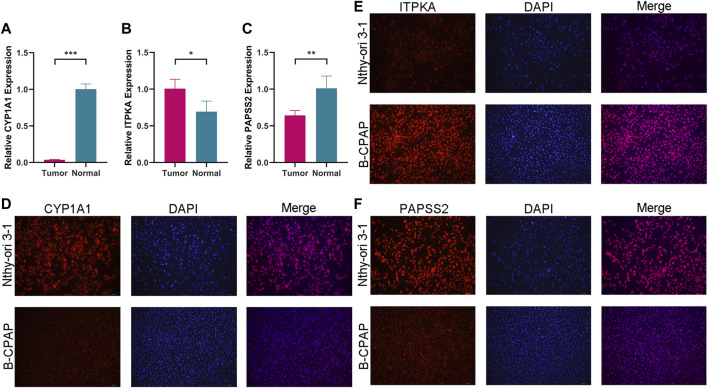
The expression levels of CYP1A1 **(A)**, ITPKA **(B)** and PAPSS2 **(C)** in tumor and normal cells. **(D**–**F)** Immunofluorescence staining of normal thyroid cells and PTC cells to further confirm differential expression.

Comparing tumor cells (B-CPAP cell line) and normal cells (Nthy-ori 3-1 cell line), the expression levels of model genes (*PAPSS2*, *ITPKA* and *CYP1A1*) were as expected. The consequences of the qRT-PCR demonstrated, the expression of *PAPSS2* and *CYP1A1* in PTC cells was lower than that of normal thyroid follicular cells, and the expression of *ITPKA* was the opposite (Figures 11A–C). Immunofluorescence staining of the cells confirmed the results above (Figures 11D–F).

### Pan-cancer analyses related to mutation of model genes

**FIGURE 12:**
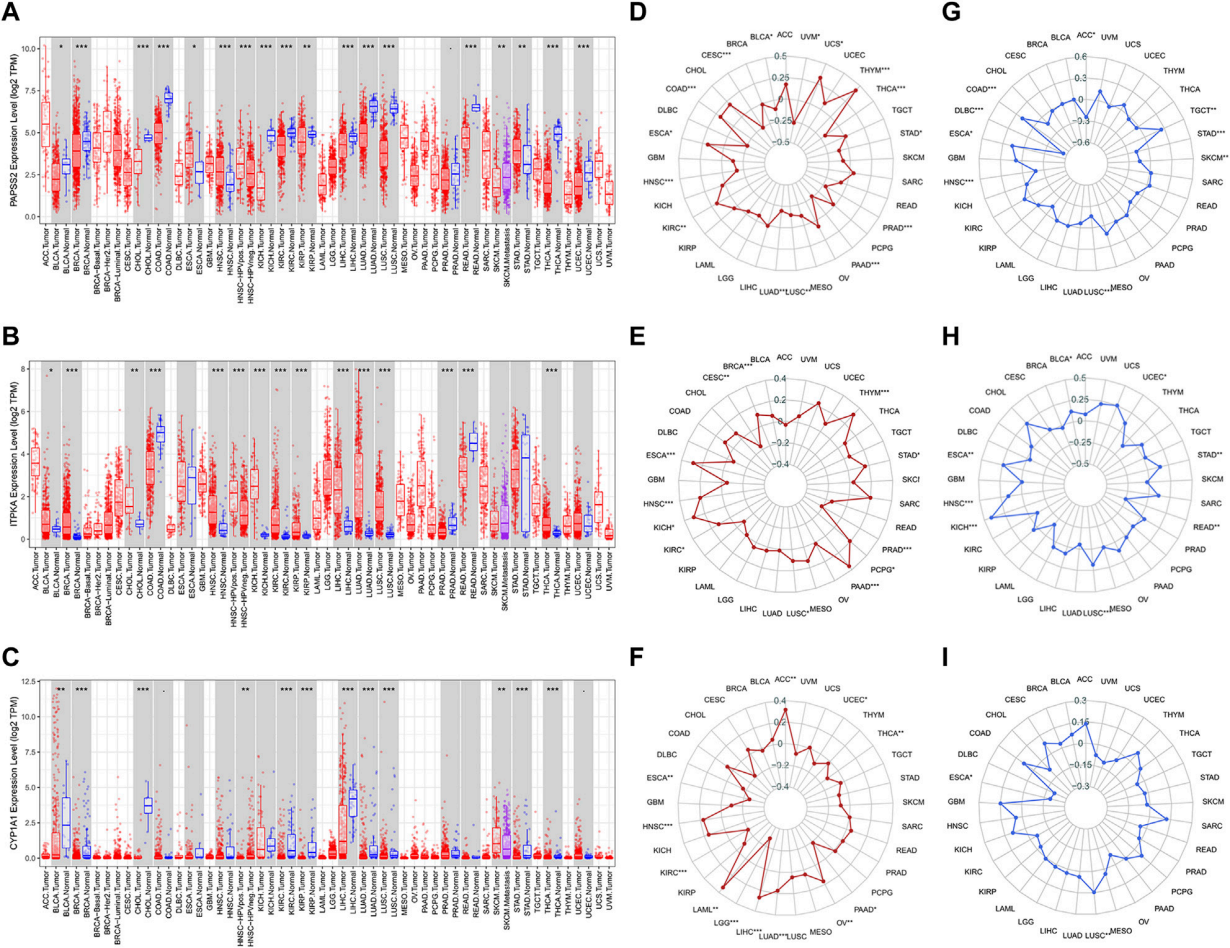
The value of model genes in pan-cancer analyses. **(A**–**C)** The mRNA expression differences of three model genes between normal and cancer tissues in pan-cancer datasets. **(D**–**F)** Correlation analyses of model gene expression and TMB. **(G**–**I)** Correlation analyses of model gene expression and MSI.

TIMER was used to visualize the mRNA expression of model genes. As was displayed in the boxed plots (Figures 12A–C), mRNA expression levels of *PAPSS2*, *ITPKA*, and *CYP1A1* had the most statistically significant differences (*p* < 0.001) in multiple normal and cancer tissues, such as invasive breast carcinoma (BRCA), kidney renal clear cell carcinoma (KIRC), liver hepatocellular carcinoma (LIHC), lung adenocarcinoma (LUAD), lung squamous cell carcinoma (LUSC), and thyroid carcinoma (THCA). Then, using radar charts, we examined the relationships between model gene expression and TMB and MSI levels in pan-cancer (Figures 12D–I). TMB demonstrated differences (*p* < 0.05) related to the expression of model genes in pancreatic adenocarcinoma (PAAD), kidney renal clear cell carcinoma (KIRC), head and neck squamous cell carcinoma (HNSC), and esophageal carcinoma (ESCA). In lung squamous cell carcinoma (LUSC) and esophageal carcinoma (ESCA), MSI depicted differences (*p* < 0.05) associated with the expression of model genes. Pan-cancer exploratory research may be useful for future research.

## Discussion

Thyroid carcinoma is the most common malignant endocrine disorder, with an increasing incidence recently, owing primarily to advances in image detection resolution (Lin et al., 2020; Mariniello et al., 2020). In the current background of precision medicine worldwide, molecular targeted therapy has received increasing attention. Currently, novel targeted strategies are increasingly vital in treating thyroid carcinoma, particularly suitable for aggressive thyroid tumors (Wang et al., 2019). Metabolic cancer disorders have attracted large-scale notice over the past few years (Huang et al., 2020). Epidemiological investigations indicate a link between carcinogenesis and decompensation, which backs an association among the increased risk of cancer and metabolic syndrome, inflammation, obesity, and insulin resistance (Pucci et al., 2019). However, their direct relationship regarding cancer prognosis and metabolic pathway expression remains mysterious. It is challenging to assess pathways as a single entity for survival analysis out of polygenic metabolic pathways (Zhang et al., 2020). For this reason, we built a risk model for foreseeing the survival of THCA patients with greater accuracy and efficiency. Our current research can prove that the risk score of the metabolism-related model is a high-stakes and prognostic factor independent of other clinical features.

3′-Phosphoadenosine 5′-Phosphosulfate synthase 2 (*PAPSS2*) is the significant enzyme to produce PAPS, which is the sulfate source in mammals and is created from ATP and inorganic sulfate. The defection of *PAPSS2* can lead to alarming bone development diseases, containing malformation, spondyloepimetaphyseal dysplasia, hepatocellular carcinoma, estrogenic hormone disorder, and so on (Zhang et al., 2022). Reports related to *PAPSS2* and cancer have preliminarily suggested the research value of *PAPSS2*. Evidence suggests that high expression of *PAPSS2* was linked to decreased prevalence of human colitis and colon cancer, with *PAPSS2* protecting against colitis and associated colonic carcinogenesis over intestinal sulfomucin and furtherance of bile acid homeostasis by way of the PAPSS2–PAPS–sulfation axis (Xu et al., 2021). Nevertheless, the enhanced *PAPSS2* is vital to push migration and metastasis of breast cancer cells (Zhang et al., 2019) and shows shorter relapse-free survival periods in cancer of the breast (Jung et al., 2016). Furthermore, we discovered an intriguing report that identified *PAPSS2* as a candidate gene for extending life span through a meta-analysis GWAS of survival to age 90 (Yerges-Armstrong et al., 2016). This may be connected with *PAPSS2*-mediated premature senescence (Jung et al., 2016). Inositol-trisphosphate 3-kinase A (*ITPKA*) is ectopically expressed in several human cancers in addition to neurons of the central nervous system and duodenum, which is important in controlling calcium signaling pathway and actin dynamics, and it may provide new options for treatment for patients (Blechner et al., 2020), such as testis, thyroid, lung, pancreas, breast, prostate, colon, liver and breast cancer so on (Windhorst et al., 2017). Upregulated *ITPKA* expression discovered in patients with breast cancer, it may serve as an independent prognostic marker in cancer of the breast (Zhang et al., 2021b). Besides its demonstrated association with carcinoma prognosis, *ITPKA* promotes cancer cell growth, invasion, and migration in renal cell carcinoma (Zhu et al., 2020) and lung adenocarcinoma (Guoren et al., 2020). Based on the functional activities of increasing invasive migration of tumor cells, *ITPKA* might become an innovative target for inhibiting invasion and metastasis of primary tumors (Windhorst et al., 2010). Subfamily 1 of the Cytochrome P450 family Cytochrome P450 enzymes (P450s or CYPs), a unique family of heme proteins containing ferrous ion (Fe2+) and functioning as oxygenases, are linked to the pathogenesis of several diseases, including primary congenital glaucoma (buphthalmos), inflammatory disease, and cancers (Kwon et al., 2021). It has been published that *via* preventing PTEN and stimulating β-Catenin and Akt pathways, the AhR/CYP1A1 signaling pathway affected the phenotypes of breast cancer stem cells, such as growth, development, renewal, and chemoresistance (Al-Dhfyan et al., 2017). Recent research has covered the genetic polymorphisms of *CYP1A1* carried weight with the risk of developing cancers containing lung cancer (Kudhair et al., 2020), upper digestive tract cancer (Tian et al., 2019; Zhao et al., 2019), and thyroid carcinoma (Bufalo et al., 2006; Figlioli et al., 2016). In thyroid carcinoma patients, *CYP1A1* could serve as a prognostic marker to point to the grade of tumor severity, with the *CYP1A1* gene expression associated with tumor size, the presence of metastasis, and advanced clinical stage (GallegosVargas et al., 2016).

Our research obtained 201 differential genes through the extraction and differential analysis of 944 metabolic genes. We preliminarily identified prognostic-related genes among differential genes with univariate Cox analysis. Following the results of LASSO Cox regression analysis and multivariate Cox regression analysis, three genes were selected to construct a prognostic model of metabolism-related genes. The prognostic value of the three-gene risk model was estimated in the training dataset and validated in a test dataset. The survival curves demonstrated that the overall survival rate of the low-risk cohort was higher than that of the high-risk cohort. The AUC of the ROC curve in the training and test sets both demonstrated the effectiveness of risk models for prognostic prediction. With clinical features and risk scores involved in the multivariate Cox analysis, the risk score achieved from the metabolism-related model could be an independent predictor. To illuminate the bond between infiltrating immune cells and risk score, we sized up the infiltration of tumor immune cells between two high-stakes and low-stakes groups of patients. In two sets, the infiltration levels of plasma cells were statistically different between low-stakes and high-stakes groups. In addition, immune-related functions of parainflammation, CCR and type II IFN response with the ssGSEA algorithms were statistically different between high-stakes and low-stakes groups. By further assessing the availableness of the manufactured risk model in immunotherapy, we discovered that the expression of *IDO1*, *IDO2*, *CD80* and *CD28* were most different statistically in the two risk groups. Interestingly, the advancement of the pathological staging of PTC was linked to the abnormal expression of diverse immune checkpoints (Yang et al., 2021). Then, we found that the low-risk groups demonstrated high TMB. Discoveries on mutational load indicate its function of foretelling survival in human cancers. As the TMB of a patient gets high, the hazard ratio of death drops in most cancers (Samstein et al., 2019). Therefore, the results of the TMB analysis are consistent with our previous study of overall survival in different risk groups.

In addition to the discussion of immune value and mutation value, we found that the drug sensitivity of patients with different risk statuses to antitumor therapy is not the same. In our study, high-risk patients were more sensitive to Veliparib and Afatinib. It has been reported that the PARP inhibitor Olaparib in association with an oncolytic virus named dl922-947, had a synergistic antitumoral effect on anaplastic thyroid carcinoma (Passaro et al., 2015). As a PARP inhibitor, Veliparib may have the same effect. Huang et al. discovered that Afatinib, an EGFR-targeting drug, had significant anticancer activity in SW579 cells, an EGFR-expressing human ATC cell line, including growth inhibition and cell death occurrence (Huang et al., 2018). The existing research base is consistent with our research. In differentiated thyroid carcinoma, the expression and functional integrity of NA^+^/I^−^ symporter (*NIS*/*SLC5A5*) are decisive for the ability of concentrating iodine (Castro et al., 2001; Wang et al., 2013). The NIS expression in the high-risk group was higher than that in the low-risk group, which indicated the response of radioactive iodine (RAI) therapy may be better in high-risk group. This finding may provide a reference for predicting the sensitivity of RAI therapy in DTC, but we should know the changes of other molecules that regulate intracellular metabolism also have effects on the effectiveness of RAI therapy (de Morais et al., 2018).

Excitingly, there are few studies on metabolic-related genes and THCA, and our research may be of great relevance for the future. Unfortunately, our research had unresolved regrets, such as using only a single database for data analysis. Although the sample size was not small and could meet statistical needs, we originally planned to use another database for external verification. However, we could not complete the expected plan due to the lack of data in other databases. So we randomly split samples in a one-to-one ratio to meet the reasonable verification required by the plan. Furthermore, more basic experiments should be performed to better investigate the feasibility of immunotherapy in thyroid carcinoma. Besides, we look forward to more extensive follow-up studies to understand the clinical value of the risk model in thyroid cancer.

## Conclusion

This research built an innovative risk model of three MRGs for cases with THCA. As stated in the research results, this risk model was expected to become an effective and independent prognostic point of reference for THCA. Furthermore, our research provided basic insights on the association between MRGs and survival and analyzed immune status, mutation and therapy sensitivity of patients at different risks. In conclusion, this risk model is predictive of patient survival, and three genes selected are a potential tool to understand more thyroid tumor genesis and possible treatment.

## Data Availability

Publicly available datasets were analyzed in this study. This data can be found here: Genomic Data Commons (https://portal.gdc.cancer.gov/); the UCSC Xena website (https://xena.ucsc.edu/).
