# Supplementary material for: A metabolism-related gene signature for predicting the prognosis in thyroid carcinoma
Source: Front Genet. 2023 Jan 4;13:972950. doi: 10.3389/fgene.2022.972950 (PMC9846547; doi:10.3389/fgene.2022.972950)
Supplement: Supplementary file 1 [file DataSheet1.docx]

Supplementary Material
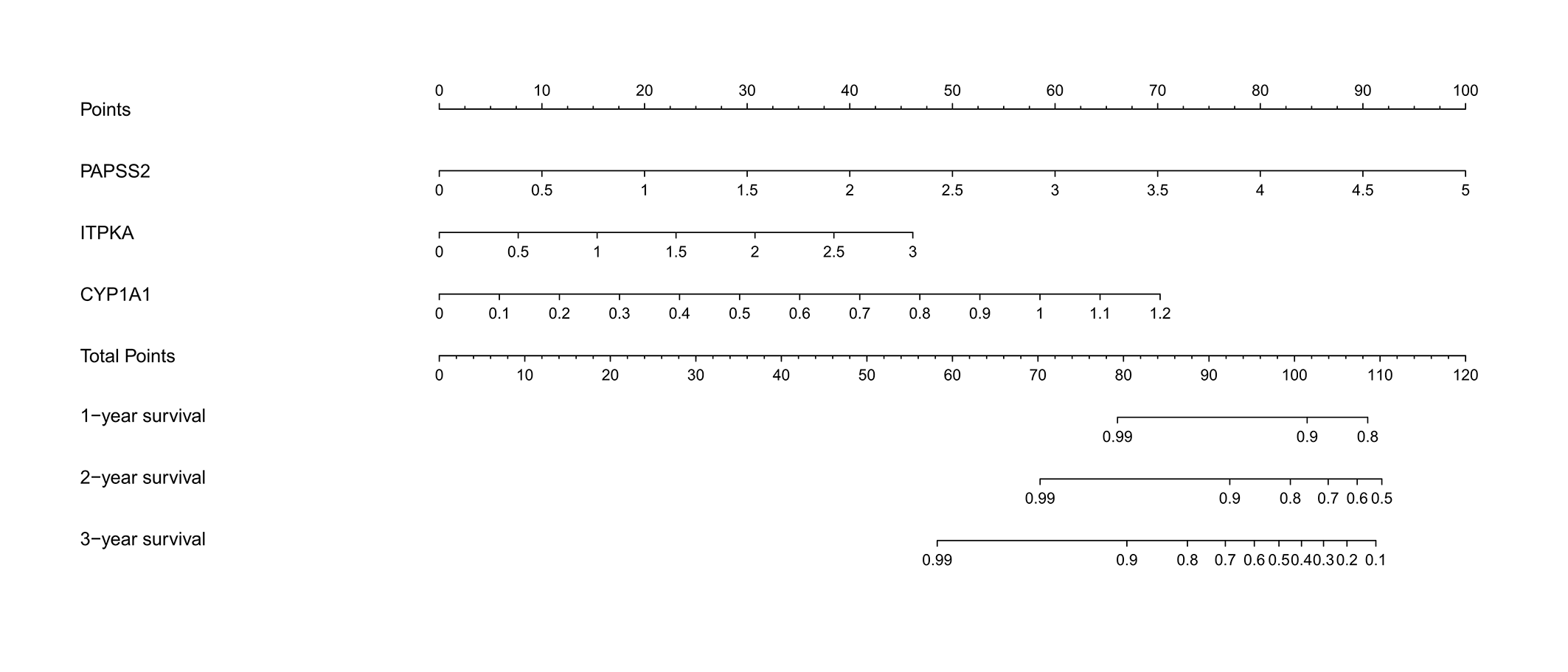


**Supplementary Figure S1.** A nomogram based on the three metabolism-related genes.


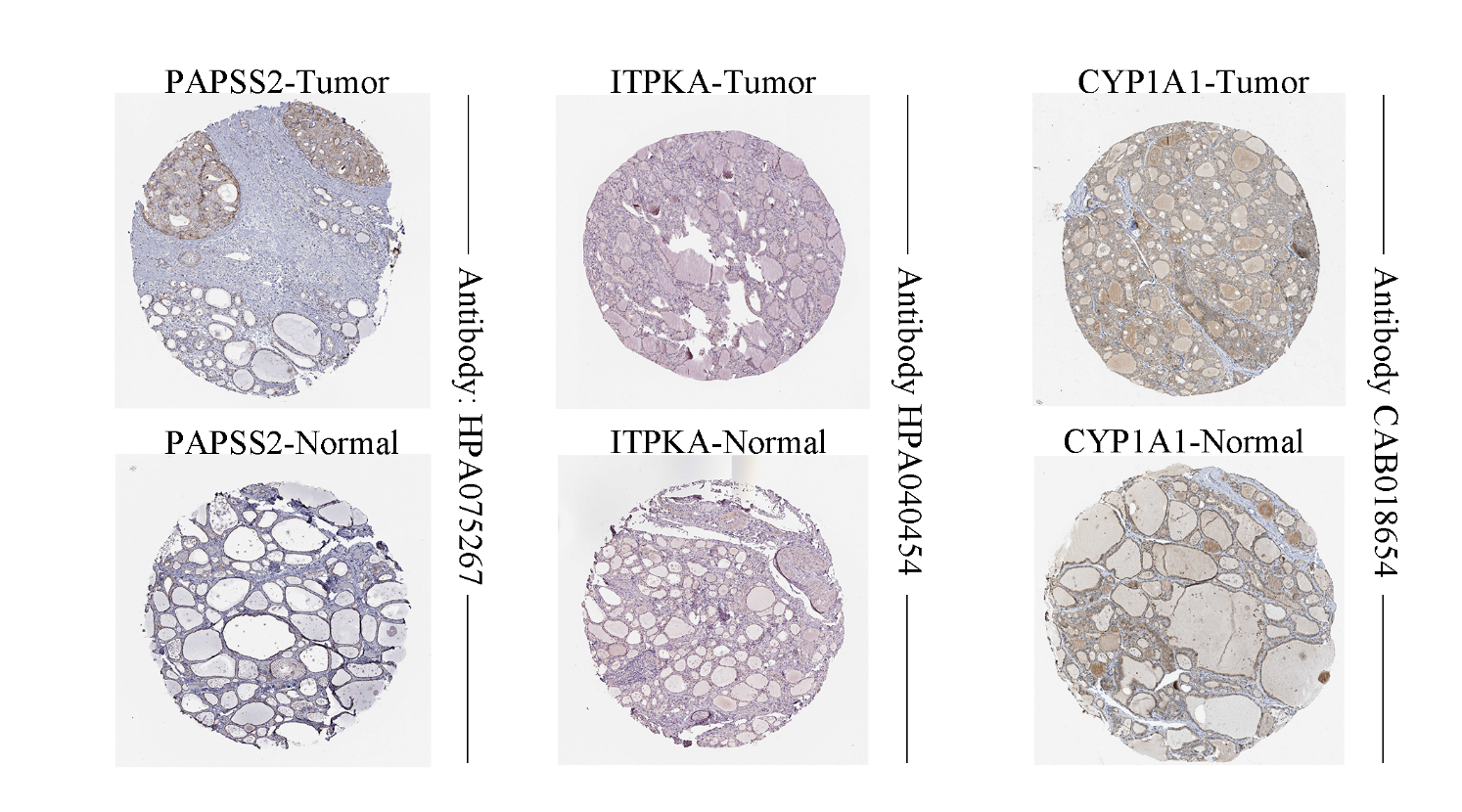


**Supplementary Figure S2.** IHC analysis of the protein expression of three model genes including thyroid carcinoma and thyroid gland in HPA.


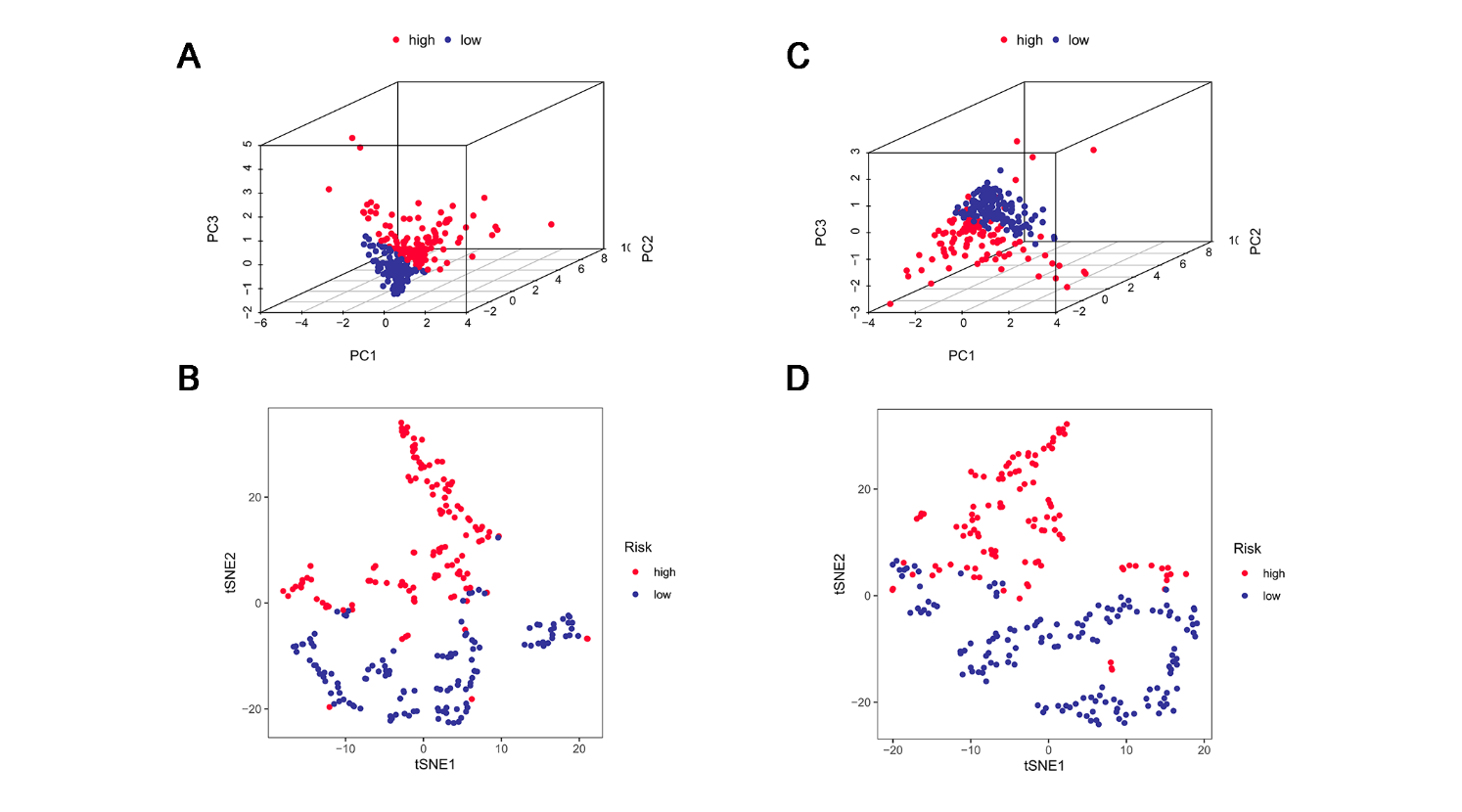
 **Supplementary Figure S3.** The affirmation of precision of the assortment. **(A)** PCA in three dimensions plots in the training cohort. **(B)** The t-SNE plot in the training cohort. **(C)** PCA in three dimensions plot in the test cohort. **(C)** PCA in three dimensions plot in the training cohort. **(D)** The t-SNE plot in the test cohort.


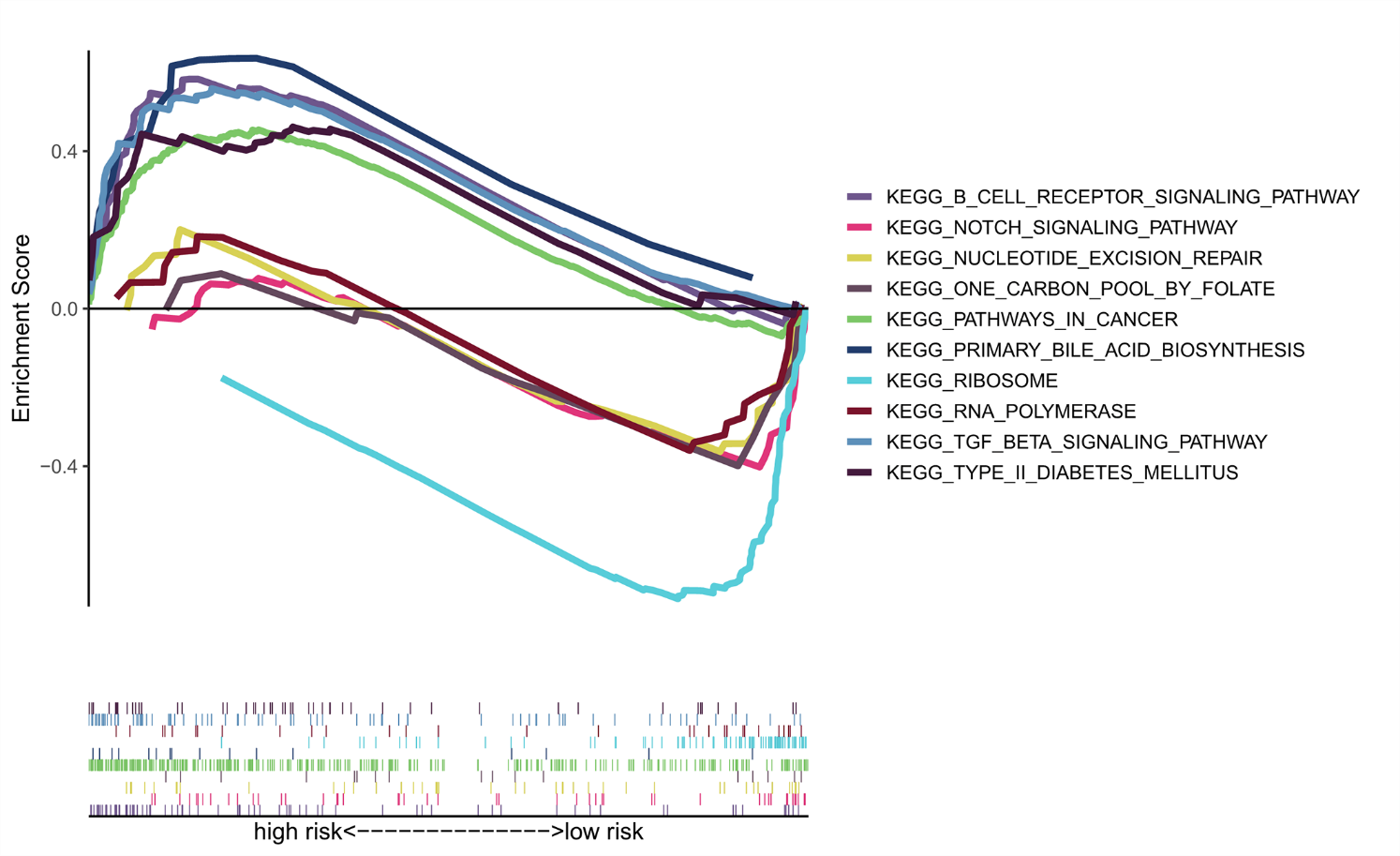


**Supplementary Figure S4.** Ten important pathways between two risk cohorts in GSEA enrichment analysis.

**
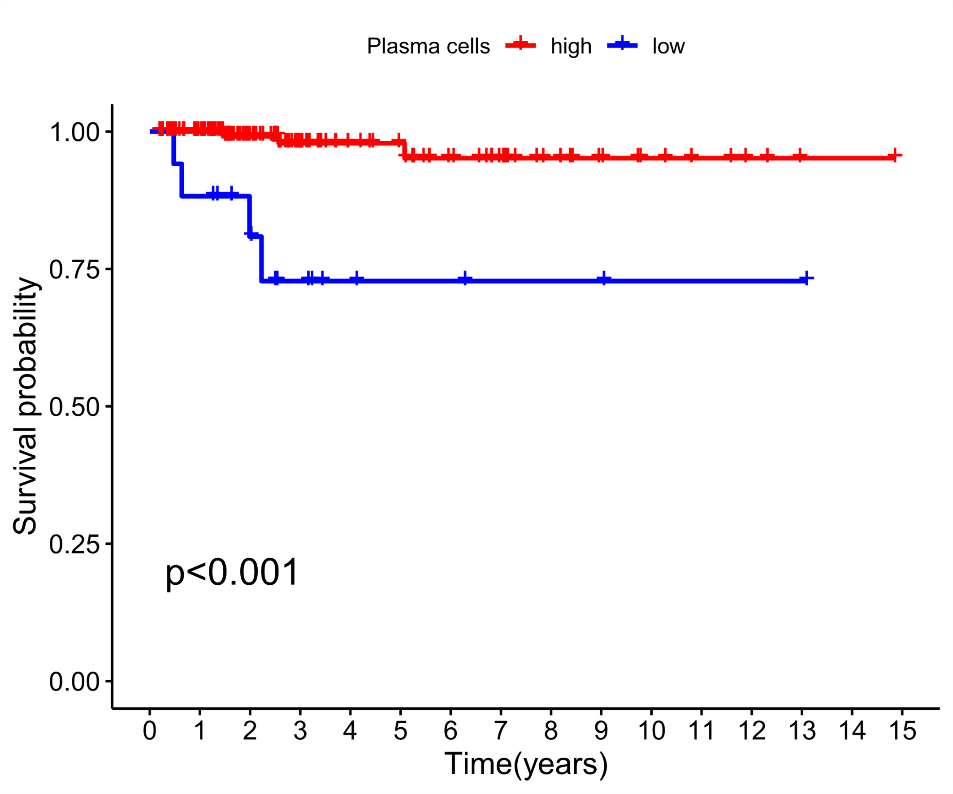
**

**Supplementary Figure S5.** Survival curves for high and low plasma cell scores based on TCGA-THCA dataset.

**
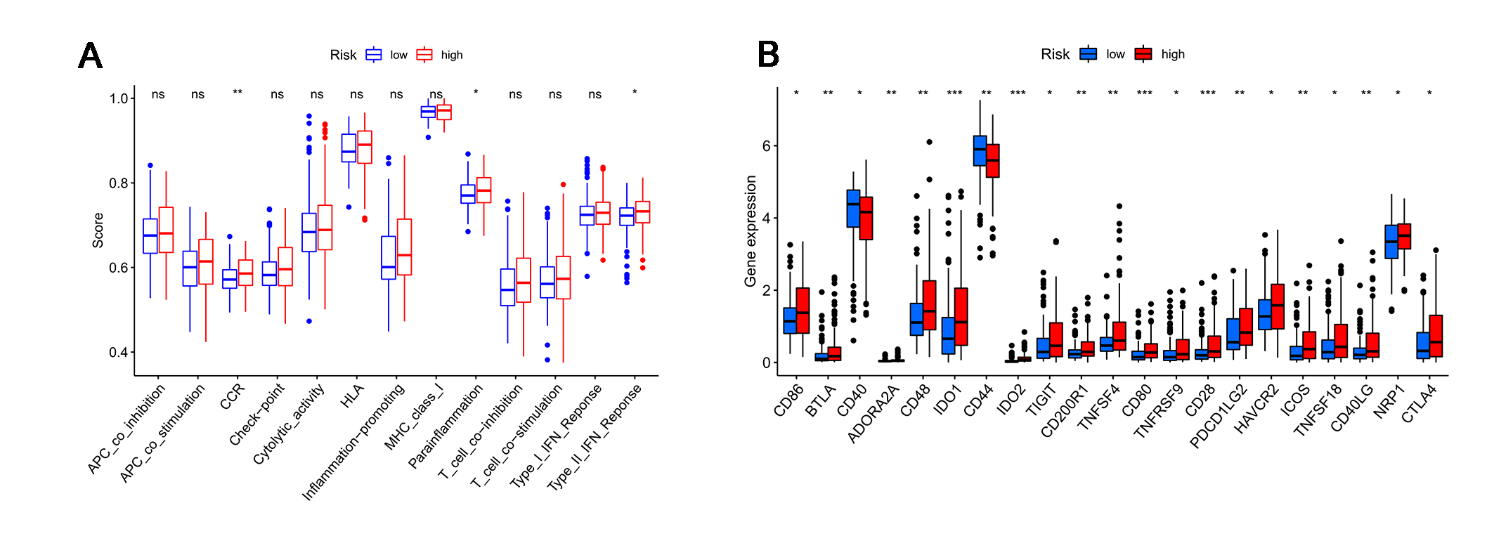
**

**Supplementary Figure S6.** Analyses of immune-related functions and immune checkpoints between risk cohorts. **(A)** The comparison of immune-related functions based on the ssGSEA algorithms. **(B)** Gene expression differences at immune checkpoints in the TCGA-THCA dataset. The statement of significance: *p < 0.05, **p < 0.01, ***p < 0.001.

**
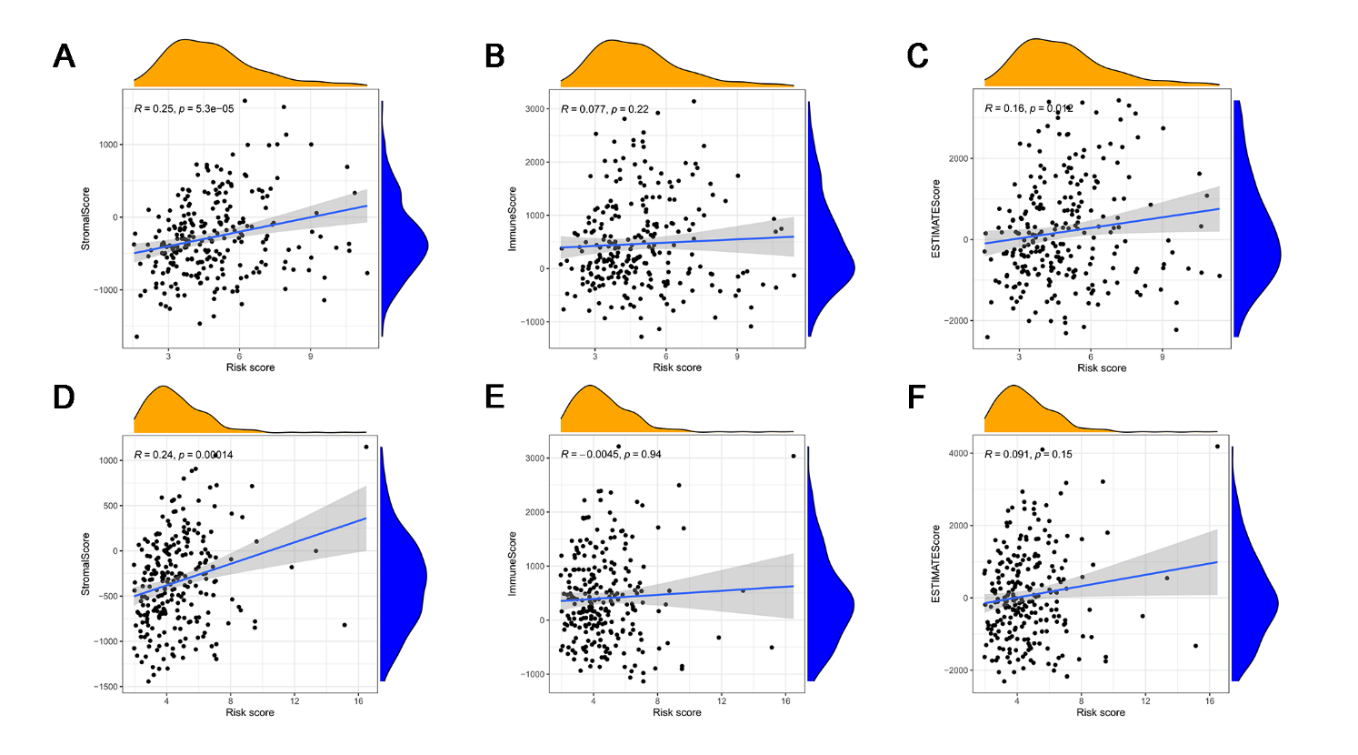
**

**Supplementary Figure S7.** Comparison of risk scores and cellular scores about the tumor microenvironment based on the ESTIMATE algorithms. **(A-C)** The correlation of calculated scores and risk scores in the training set. **(D-F)** The correlation of calculated scores and risk scores in the test set.


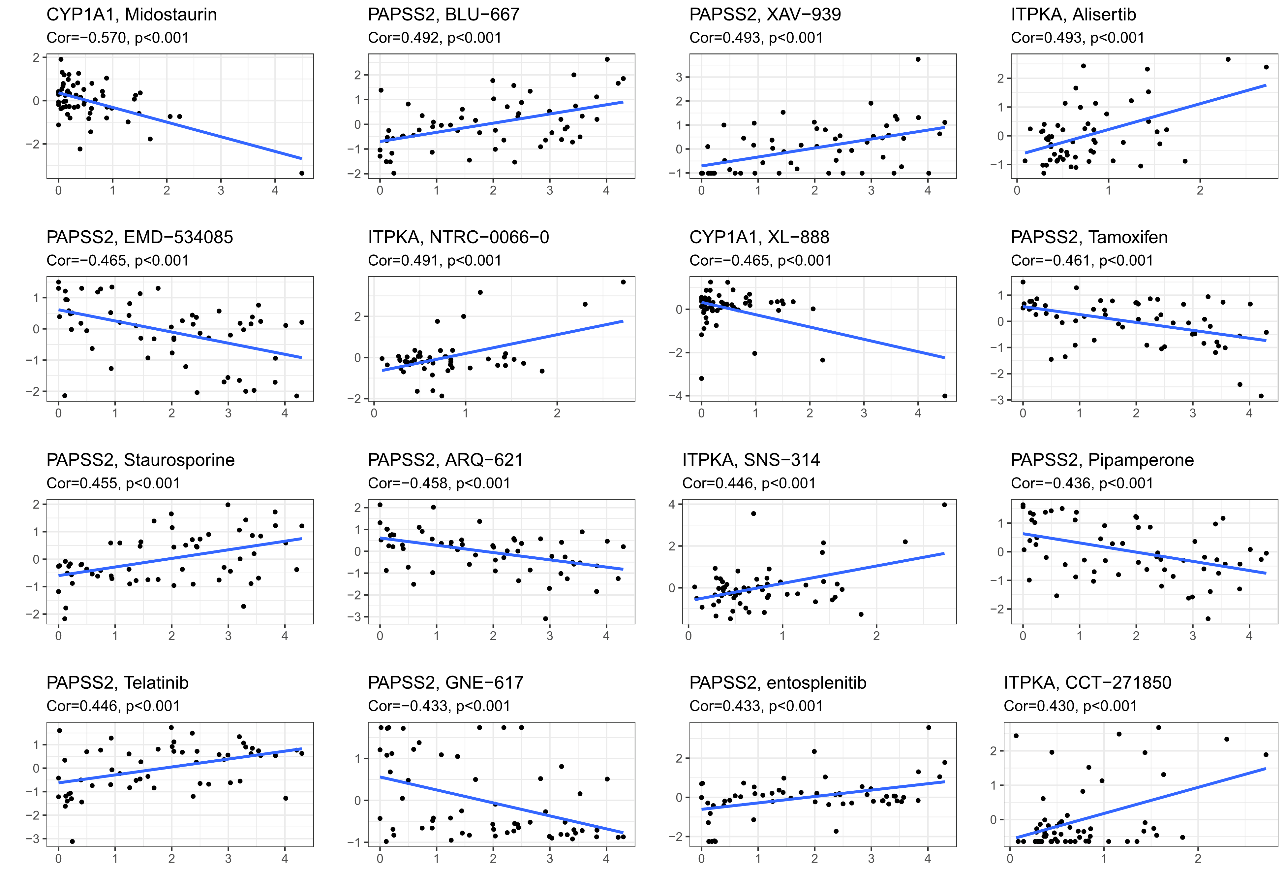


**Supplementary Figure S8.** Correlation prediction between gene expression and chemotherapeutic drug sensitivity based on the CellMiner database.


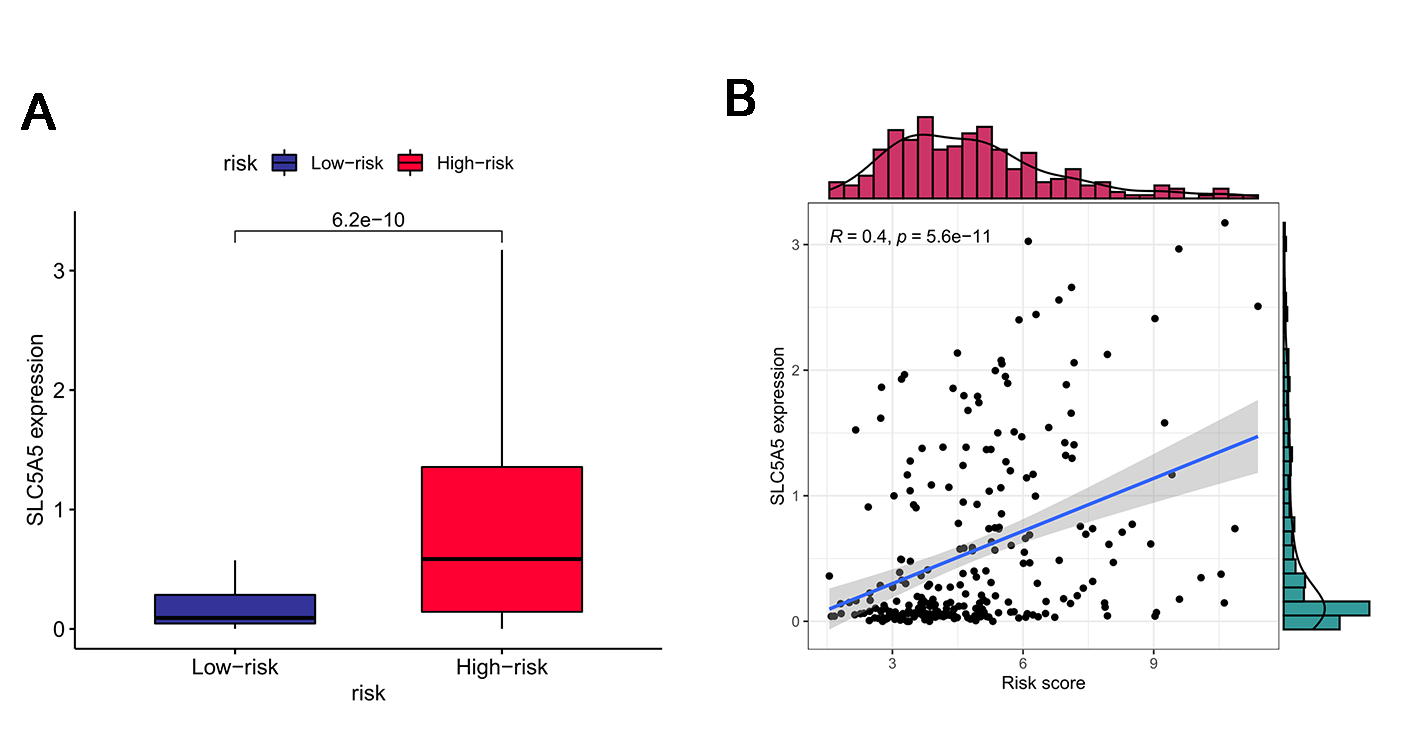


**Supplementary Figure S9.** Preliminary prediction of response to radioiodine therapy.

**Supplementary Table S1. The sequences of the primers**

| **Premier** | **Sequences (5’-3’)** |
| --- | --- |
| PAPSS2 | GAGCAGGAATAAGAGAGGGCAAGTG  AGGGATGGCATGGGAGACAAGG |
| ITPKA | GCAGCACCGACTTCAAGACTACG  GTTCAGATACCGCCTCAGCACTTC |
| CYP1A1 | TTCGCTACCTACCCAACCCTTCC  GTAGTGCTCCTTGACCATCTTCTGC |

**Supplementary Table S2. Descriptive statistics of pathological types in the training group**

|  | **N** | **Mean** | **Std. deviation** | **Std. Error** | **95% CI** | **minimum** | **maximum** |
| --- | --- | --- | --- | --- | --- | --- | --- |
| Papillary adenocarcinoma, NOS | 181 | 4.961 | 2.021 | 0.150 | 4.665-5.258 | 1.551 | 11.393 |
| Papillary carcinoma, follicular variant | 50 | 4.469 | 1.528 | 0.216 | 4.035-4.904 | 1.597 | 8.072 |
| Papillary carcinoma, columnar cell | 17 | 4.559 | 1.439 | 0.349 | 3.819-5.298 | 2.010 | 7.597 |
| Total | 248 | 4.835 | 1.902 | 0.121 | 4.597 | 5.073 | 1.551 |

Illustration: Three pathological types were excluded from statistical analysis due to small sample size. 4 excluded samples included one case of follicular carcinoma (minimally invasive), two cases of nonencapsulated sclerosing carcinoma, one case of oxyphilic adenocarcinoma.

**Supplementary Table S3.** **Analysis of variance (ANOVA)**

| **Source** | **SS** | **df** | **MS** | **F** | **P** |
| --- | --- | --- | --- | --- | --- |
| Groups | 10.884 | 2 | 5.442 | 1.510 | 0.223 |
| Error | 883.086 | 245 | 3.604 |  |  |
| Total | 893.971 | 247 |  |  |  |

**Supplementary Table S4. Multiple Linear Regression (MLR)**

| **Model** | **b** | **Std.Error** | **Beta** | **t** | **Sig.** | **Collinearity Statistics** | | **R^2^** |
| --- | --- | --- | --- | --- | --- | --- | --- | --- |
|  |  |  |  |  |  | **Tolerance** | **VIF** |  |
| Constant | 4.961 | 0.141 |  | 35.158 | 0.000 |  |  | 0.012 |
| B | -0.492 | 0.303 | -0.104 | -1.623 | 0.106 | 0.981 | 1.019 |  |
| C | -0.403 | 0.482 | -0.054 | -0.837 | 0.404 | 0.981 | 1.019 |  |

Illustration: Constant was papillary adenocarcinoma (NOS). B was papillary carcinoma (follicular variant). C was papillary carcinoma (columnar cell).
